# Platelet-Rich Plasma Plus Microneedling for Scar Management: Introduction to an Innovative Technique and Initial Results From 107 Patients

**DOI:** 10.7759/cureus.81294

**Published:** 2025-03-27

**Authors:** Gioia S Kouthoofd, Ester M Peters, Jean-Bart Jaquet

**Affiliations:** 1 Department of Plastic Surgery, Amphia Hospital, Breda, NLD; 2 Department of Plastic and Reconstructive Surgery, Maasstad Hospital, Rotterdam, NLD

**Keywords:** micro-needling, platelet-rich plasma, platelet-rich plasma (prp), prp, scar

## Abstract

Background

Scars are known to negatively impact patients’ quality of life or even disable them. A broad spectrum of different scar treatments is used in clinical practice. Platelet-rich plasma (PRP) and microneedling are promising treatments that enhance tissue regeneration. The aim of this study was to evaluate the safety, clinical effects, and patients’ experience of a combination treatment consisting of a series of PRP plus microneedling.

Methods

One hundred seven patients with scars, with various etiology and scar age, were included in our study. PRP had been prepared using the ACP double syringe system and was injected with high pressure into the dermis of the affected tissue, using the U225 meso injector as an injection and microneedling device. This treatment was applied three times at four-week intervals. On the days of treatment and at the 4-week follow-up, patient records were collected and clinical investigations and interviews were performed to assess clinical effects, adverse effects, and patient’s experience.

Results

The results of this retrospective study indicate that 23 patients (21.5%) reported softer and more elastic scar tissue, 8 patients (7.5%) experienced improved mobility and functionality, 6 patients (5.6%) showed an overall healthier skin appearance, and 8 (7.5%) patients observed an improvement in color.

Adverse effects reported were mild and only temporary. Twenty-three (21.5%) patients felt stressed before the treatment, and 12 patients (11.2%) reported tenderness during the first treatment of which two rated it as mildly painful. After the first treatment, two patients (1.9%) reported transient itchiness. Serious adverse effects did not occur.

Conclusions

PRP plus microneedling can enhance the pliability, mobility, and color of different scar types and scar ages. Overall, the treatment was easily feasible, well-tolerated and safe. Therefore, it can be considered a promising new treatment modality for scar management.

## Introduction

Scars are known to negatively impact patients’ quality of life, due to tenderness, pain, and psychosocial factors and evidently, they can lead to physical disabilities in the case of contractures depending on location and size [1-3]. Scar management consists of various treatment modalities leading to beneficial results regarding pain, pruritus, pigmentation, pliability, surface area, and thickness [4]. Application of platelet-rich plasma (PRP) is a promising innovative autologous treatment modality in scar management and is already used in numerous medical fields, among others in cardiothoracic, maxillofacial, neuro and orthopedic surgery, sports medicine, urology, ophthalmology, aesthetic dermatology, plastic surgery and for wound healing treatments [5].

The exact mechanism and effects of PRP on scar tissue are not yet fully understood. PRP contains a high number of thrombocytes, which are known for their contribution to the hemostatic processes and tissue regeneration. Activated platelets release growth factors and cytokines contributing to wound healing, immune activation, mitogenesis, angiogenesis, and macrophage activation. Numerous growth factors are contained in PRP, among them platelet-derived growth factor (PDGF), transforming growth factor beta 1 and 2 (TGF-b), vascular endothelial growth factor (VEGF), insulin-like growth factor (IGF-1), platelet factor (PF), stromal cell-derived factor (SDF-1), epidermal growth factor (EDF), and fibroblast growth factor (FGF) [6-9].

TGF-b mediates long-term healing mechanisms since it affects mainly fibroblasts, stem cells, and pre-osteoblasts, which can synthesize and excrete TGF-b on their own [6-8]. Dense bodies contain adenosine diphosphate (ADP) and serotonin [10]. ADP leads to conformational change in the fibrinogen receptor and to aggregation and activation of platelets. PRP has been shown to attenuate fibrosis, increase the production of procollagen type I, and decrease apoptosis. Moreover, it induces muscle and endothelial cell proliferation [7,8].

PRP injected in the dermis and subdermis resulted in cellular changes in soft-tissue augmentation, angiogenesis, and stimulation of subdermal adipocytes [8].

Microneedling is frequently used for the treatment of scars. It triggers skin repair mechanisms and increases dermal collagen and elastin deposition [11-15].

Microneedling at a depth of 3 mm reaches the papillary and reticular dermis, while the stratum corneum, the epidermal barrier, is preserved. So, no scarring is expected in this procedure [11]. In facial treatments microneedling in combination with PRP has been shown to enhance percutaneous collagen induction [11,16]. Moreover, significantly better improvement of acne scars was reported with PRP plus microneedling vs. microneedling alone [17].

PRP enhances sustainable tissue regeneration, potentially even after the chronic phase of wound healing, so positive effects on scar tissue may be anticipated as well. The combination of microneedling and PRP has already been shown to be effective in acne scars, with minimal side effects [17]. Frequently microneedling pens or rollers are used. Microneedling increases the absorption of topically applied products [11]. Nevertheless, a lot of the topically applied substance gets lost. Therefore, we decided to use a pneumatic injector as an injection and microneedling system. The aim of our study is to evaluate the safety, the clinical effects, and the patients’ experience after a series of simultaneous PRP injections and microneedling for the treatment of scars.

This article was previously presented as a meeting abstract at the 20th European Burns Association Congress & 42e SFB Congress in Nantes, France, on September 8, 2023.

## Materials and methods

Data collection

All patients who underwent scar treatments at the Plastic Surgery department of the Maasstad Hospital (Rotterdam, The Netherlands) between March 2021 and September 2022 were included in this retrospective study. Approval from the local ethics commission was obtained for this study (Approval number: L2023109), the principles outlined in the Declaration of Helsinki were followed, and consent was obtained from all patients. Patient demographics, including age and sex, were evaluated. Moreover, analyzed clinical data included the etiology of the injury which resulted in the scar, the location, and the age of the scar. Data from clinical investigations, interviews, and other patient records were used to evaluate patient’s experiences, adverse effects, and clinical outcomes. All observations were conducted on the treatment days and at the end of the treatment course (four weeks after the last treatment) routinely.

Platelet-rich plasma plus microneedling procedure

Treatments were performed by a qualified doctor or skin therapist. Fifteen milliliters of whole blood were collected with an ACP Double Syringe system (Arthrex Inc., Naples, FL, USA). Then the syringe was spun in a bench-top centrifuge (Rotofix 32 A centrifuge, Andreas Hettich GmbH & Co. Kg, Tuttlingen, Germany) for 5 minutes at 1500 RPM to obtain the PRP. No anticoagulantia was used in this process. The inner syringe containing the PRP was twisted out and placed in the U225 meso injector (Needle Concept, Biarritz, France), which has a built-in compressor system [18]. The diameter of the needles used for injection was 0.25 mm (31 G) and the length was 12 mm. The depth of injection was set at 1-4 mm (until pain was experienced or blood was recognized). At the beginning of the treatment slow injections with a higher volume of PRP were given into the affected area, followed by a multitude of continuous quick injections with less volume (Video 1). Residual PRP was massaged into the treated area. Patients were instructed to not wash the treated area for at least 12 hours. This procedure was repeated three times with four-week intervals in between treatments.

**Video 1:** Simultaneous injection of PRP and microneedling. PRP: platelet-rich plasma

## Results

A total of 107 patients were included in this study. The mean age of the patients was 44 (18-85) years. Seventy-four (69.2%) patients were female and 33 (30.8%) were male. Various types of scars were included: Scars after burn injuries represented the largest etiology with 51.4% (55/107), followed by traumatic injuries and surgical procedures (39.3%, 42/107). Almost 9.3% (10/107) of all scars were related to other causes such as radiotherapy, necrotizing soft-tissue infections, keloid, or acne. The time between the emergence of the scar and the treatment varied between three months and 70 years. Scar characteristics are shown in Table 1.

**Table 1:** Scar characteristics.

		Burn (n = 55)	Radiotherapy (n = 1)	Trauma (n = 42)	Necrotizing soft-tissue infections (n = 4)	Keloid (n = 2)	Acne (n = 3)
Age of the scar	0-6 months	5		2			
	7-11 months	2		30			
	1-5 years	27		3	4	2	3
	6-10 years	8		7			
	11-30 years	9	1				
	>30 years	4					
Location	Head and/or neck	20		12	1	1	3
	Torso	6	1	6	2	1	
	Extremities	29		24	1		

Out of all 107 patients, 94 (87.9%) underwent three PRP treatments, and 101 (94.4%) underwent at least two PRP treatments. The full series of three treatments had not been performed in 13 patients due to the following reasons: non-compliance (5 cases; 4.7%), unreached expectations (2 cases; 1.9 %); fear of needles despite positive effects concerning thickness and color after two treatments and high satisfaction (1 case; 0.9%), other non-treatment related issues/moving away (4 cases; 3.7%), pre-existent nerve pain (1 case; 0.9%).

In two patients the first treatment triggered a reliving of the trauma that caused the scar tissue. Nevertheless, both decided to undergo subsequent treatment, since they were very satisfied with the results.

No serious adverse events occurred. Before the first treatment, 23 patients (21.5%) felt stressed, before the second treatment this rate dropped to two patients. Twelve patients (11.2%) reported mild soreness during the treatment, of which two patients experienced this as mild pain. Sixty-nine patients (64.5%) did not experience any discomfort before or during the treatment. Two of 107 patients (1.9%) reported mild itchiness directly after the treatment, which resolved on its own. No other side effects were reported.

**Figure 1:**
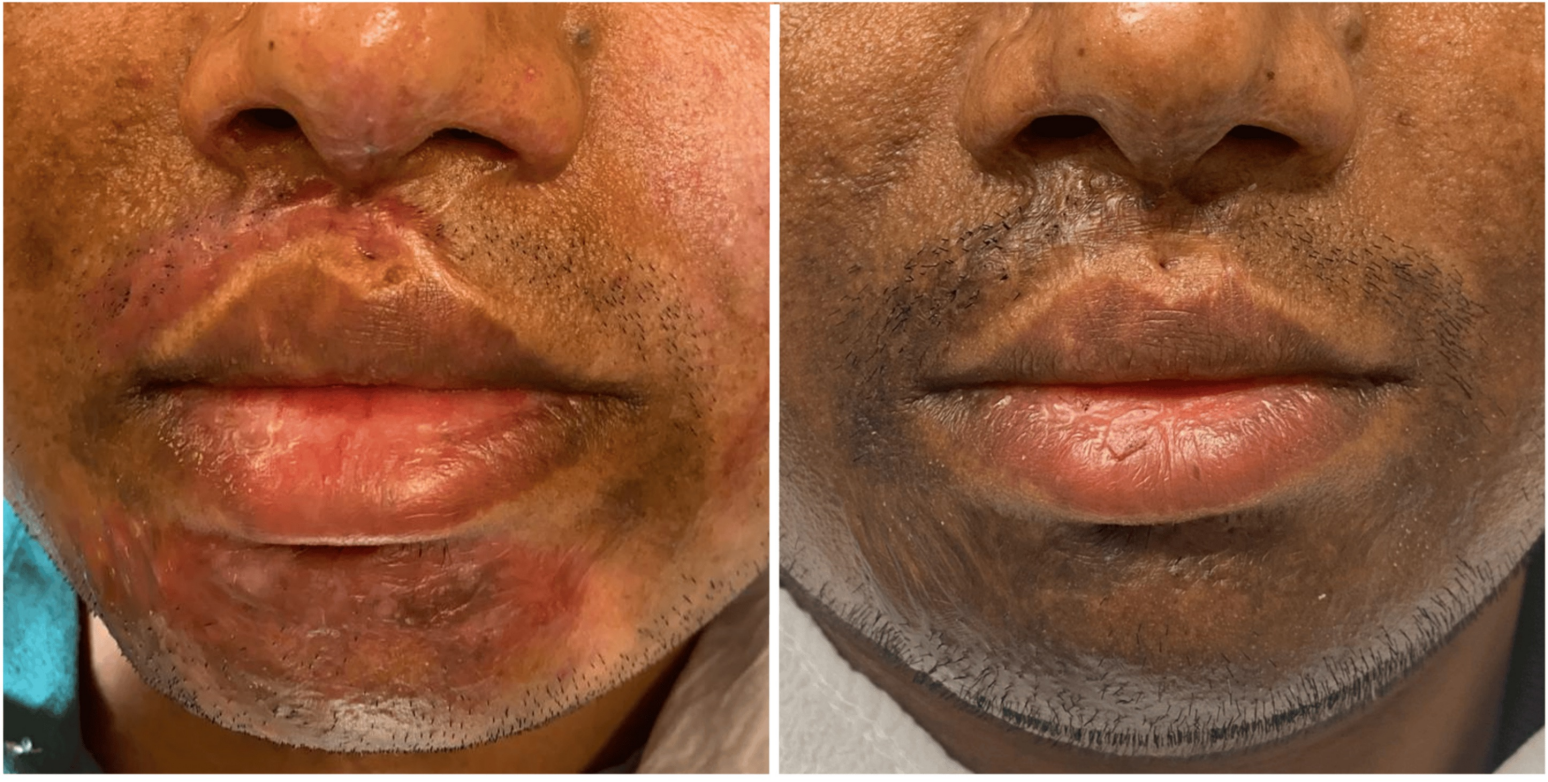
Two-year-old burn scar before (left) and four weeks after (right) three treatments with PRP plus microneedling. PRP: platelet-rich plasma

**Figure 2:**
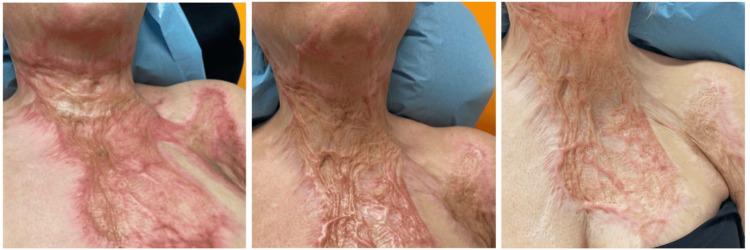
Five-month-old burn scar before (left), during (middle), and four weeks after (right) three treatments with PRP plus microneedling. PRP: platelet-rich plasma

Twenty-three patients (21.5%) reported softer and more elastic scar tissue after the first treatment. Eight patients (7.5%) experienced more mobility of the tissue, and therefore better functionality. Four patients (3.7%) reported smoother scar tissue, one patient (0,9%) reported a reduction of the surface of the scar and six patients (5.6%) had the impression of having healthier skin tissue. Eight patients (7.5%) reported an improvement in the color of the scar, and lastly, one patient (0,9%) reported decreased pain in the area of the scar tissue (Video 2 and Figures 1-2).

**Video 2:** Five-month-old burn scar after three treatments with PRP plus microneedling. PRP: platelet-rich plasma

In all patients, further improvement of the scar tissue after consecutive treatments was found (Figures 1-2). At the end of the treatment course, the scars were reported to be softer and more flexible. Additionally, the mobility of joints improved. Also, the color of the scar enhanced, and one patient reported more sensibility in the scar tissue.

During the second and third treatments, six (5.6%) and four (3.7%) patients, respectively, experienced mild soreness, which was reported to be slightly more intense compared to previous treatments. This sensation occurred during the treatment and resolved on its own. Neither soreness nor itchiness were reported after the treatment. Serious adverse events did not occur.

## Discussion

The fast and easy preparation of PRP allowed us to integrate this innovative procedure into our daily scar management routine. In total, both patients and clinicians were satisfied with the treatment experience and result. The most frequent effect was the softer, more flexible tissue of the scar. This effect subsequently resulted in the improvement of the functionality of everyday tasks. This functional improvement was mostly seen on treated scar tissue located on joints or in the abdominal area. This finding might be of high clinical relevance for patients with mobility-impairing scars. This treatment’s effect may be explained through a decreased expression of α-smooth muscle actin (α-SMA) and inhibition of the differentiation of fibroblasts into myofibroblasts [19].

Literature suggests that the combination of microneedling with PRP improves scar tissue more effectively than either PRP or microneedling alone [20]. The studies investigate different methods of PRP application, including topical and intradermal, and utilize various systems to objective the results. However, since the results in our study are based on the available data from patient records, a direct comparison between our findings and those in the literature is challenging.

In our study, we used the U225 meso injector® as an injection and microneedling system as it punctures the skin while placing an intradermal bolus of PRP, so the effect of microneedling is combined with simultaneous application of PRP. A specific feature of the U225 meso injector® is that it uses a built-in compressor system. Therefore, when the needle punctures the skin with pneumatic power, the PRP will be quickly injected into the skin allowing rapid punctures. Usually prior to microneedling numbing cream is applied [12]. We did not apply any numbing cream prior to the treatment as the rapid skin punctures minimize the injection pain anyway. This was reflected by the fact that only two patients reported mild pain during the treatment. In the first patient, a sensible area (the mandibula) was treated. In the second patient, nerve pain was already present prior to the treatment. The pneumatic feature of the U225 meso injector makes a noise that could be experienced as terrifying or stressful. Two patients were triggered to relive their trauma during the treatment. It remains unclear if the specific noise or the hospital atmosphere triggered the reaction. Anyhow, both patients decided to continue the treatment course and were satisfied with the results on the scar tissue and overall experience of the course. 38% of the few patients who retracted from the planned treatment course did so because they could not handle showing up for appointments. Patients with scars, mainly burn victims, have had a long hospitalization, some even with intensive care administration, whereby the ability to plan and keep up with hospital appointments is a challenge. Cognitive dysfunction, including executive functions, is commonly seen in patients after intensive care administration [21-23]. Creating a pleasant atmosphere for patients during their treatments is important, as burn patients frequently sustain not only physical but also psychological trauma from their injury. Therefore, negative experiences could influence their compliance and outcomes.

The majority of our patients were treated three times with PRP plus microneedling at monthly intervals, which is a common treatment protocol in plastic surgery and aesthetic dermatology. According to literature, Maximum effects are seen 12-24 weeks after microneedling. For PRP treatments, the maximal improvements of the skin were reported to occur between 1 and 3 months after the treatment lasting for up to 6 months [16,24]. Therefore, the positive effects observed in our study could be underestimated as our last follow-up was four weeks after the final treatment. For further studies, a longer follow-up period would be desirable to assess the maximum effect of PRP plus microneedling and eventually refine the treatment protocol.

In our study, four patients reported more tenderness during the consecutive treatments. This effect may be explained by the cellular changes and enhancement of the cells in the affected tissue. This finding could also mean that PRP plus microneedling can stimulate neural cells and therefore the treated area was more sensitive at consecutive treatments. This transient tenderness was well-tolerated by all patients. These observations could imply that besides the predicted cellular change in the dermis and subdermis, the neural cells and skin adnexal structures are affected as well [8, 25]. Other potential adverse effects such as post-inflammatory hyperpigmentation, transient erythema, edema, bruising, post-injection burning, or stimulation of hair growth, which have been reported in the literature, did not occur in our study [8,16,24,26]. Since no serious adverse effects occurred, PRP plus microneedling implies to be a safe treatment in scar management.

Besides the fact that our study is a retrospective cohort study without a control group, which is a limitation of our study, it should not be neglected that patient-reported outcomes are always subjective to a certain extent. The sudden attention and focus on their scar related to the treatment may have affected the way patients experienced their scar, which could influence the outcome of our study negatively [27]. In our study we did not use any scales to objectify the data, therefore all results are based on subjective self-reported, and observer-reported data. Moreover, in our study, differences in scar characteristics such as type and age of the scar tissue, or skin type were not analyzed in subgroups. Therefore, further studies with a more elaborate design would be desirable to evaluate the treatment efficacy on various types of scars.

## Conclusions

Our study revealed the positive effects of PRP plus microneedling treatment on scar tissue. Scars were softer, more pliable, and mobile after the treatment, which resulted in functional improvements. Furthermore, the scars were reportedly less visible in terms of color and surface, and in general, the skin appeared healthier. Except for minimal discomfort as post-treatment itchiness, and tenderness during the treatment, no serious adverse effects were reported. Thus, PRP plus microneedling is a promising treatment modality for scar tissue associated with minimal risks. Further studies should be conducted to test our initial findings.
